# Transcriptional analysis of the effect of exogenous decanoic acid stress on *Streptomyces roseosporus*

**DOI:** 10.1186/1475-2859-12-19

**Published:** 2013-02-21

**Authors:** Guojian Liao, Qing Liu, Jianping Xie

**Affiliations:** 1 Institute of Modern Biopharmaceuticals, State Key Laboratory Breeding Base of Eco-Environment and Bio-Resource of the Three Gorges Area, School of life sciences, School of Pharmaceutical Sciences Southwest University, Chongqing, 400715, China

**Keywords:** Daptomycin, Decanoic acid, Toxic, Tolerance, *Streptomyces roseosporus*

## Abstract

**Backgroud:**

Daptomycin is an important antibiotic against infections caused by drug-resistant pathogens. Its production critically depends on the addition of decanoic acid during fermentation. Unfortunately, decanoic acid (>2.5 mM) is toxic to daptomycin producer, *Streptomyces roseosporus*.

**Results:**

To understand the mechanism underlying decanoic tolerance or toxicity, the responses of *S. roseosporus* was determined by a combination of phospholipid fatty acid analysis, reactive oxygen species (ROS) measurement and RNA sequencing. Assays using fluorescent dyes indicated a sharp increase in reactive oxygen species during decanoic acid stress; fatty acid analysis revealed a marked increase in the composition of branched-chain fatty acids by approximately 10%, with a corresponding decrease in straight-chain fatty acids; functional analysis indicated decanoic acid stress has components common to other stress response, including perturbation of respiratory functions (*nuo* and *cyd* operons), oxidative stress, and heat shock. Interestingly, our transcriptomic analysis revealed that genes coding for components of proteasome and related to treholase synthesis were up-regulated in the decanoic acid –treated cells.

**Conclusion:**

These findings represent an important first step in understanding mechanism of decanoic acid toxicity and provide a basis for engineering microbial tolerance.

## Background

**Figure 1:**
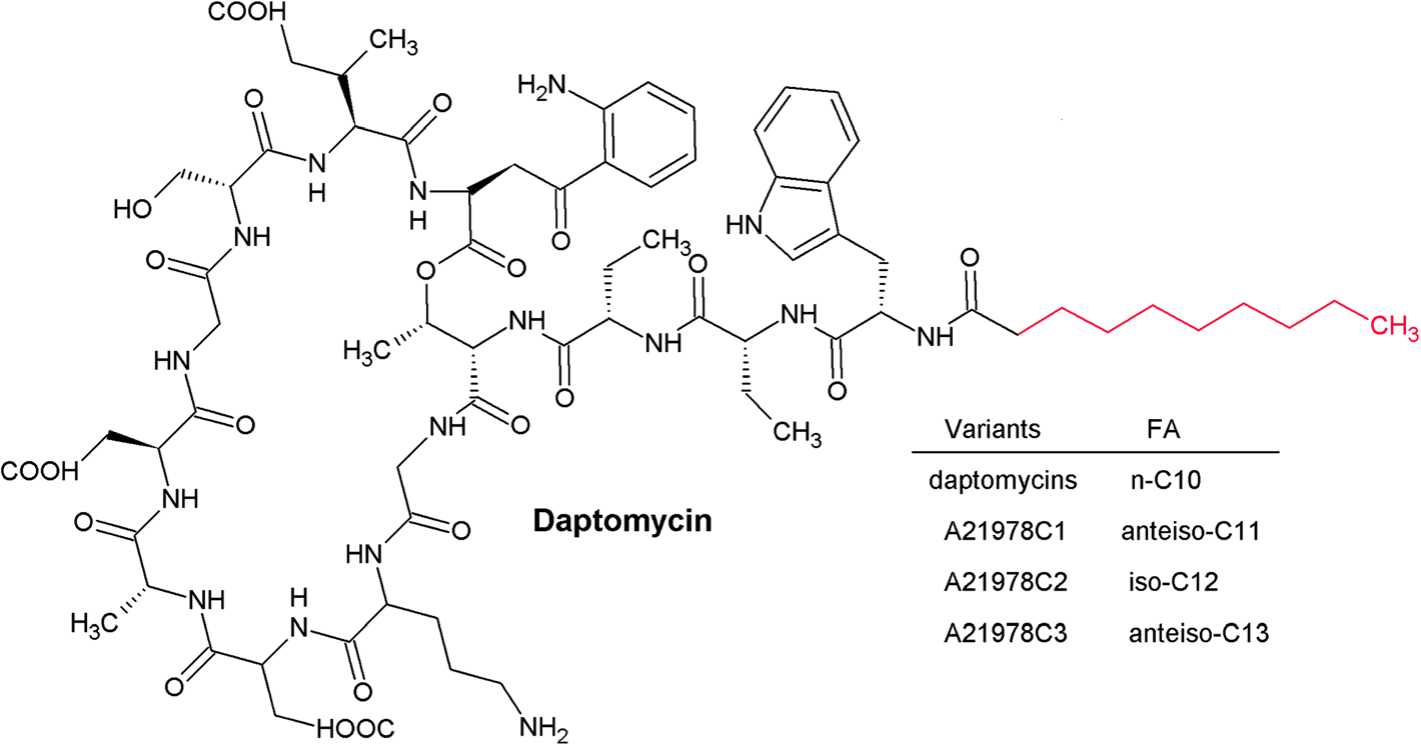
The chemical structure of daptomycin and other A21978C factors.

Daptomycin, produced by *Stretomyces roseosporus*, is a 10-membered cyclic lipopeptide showing excellent activity against Gram-positive pathogens, including methicillin-resistant *Staphylococcus aureus* (MRSA) or vancomycin-resistant *Enterococci* (VRE) [1]. Intensive efforts to improve daptomycin yield are carried out, including strain improvement as well as optimization of process conditions and growth media [2-4]. Daptomycin is the minor component of A21978C factors isolated from cultures of *S. rosoesporus *[5]. The mixture has a common cyclic peptide nucleus with different fatty acid moieties attached to N-terminal Trp (Figure 1). The addition of decanoic acid (DA) to the culture broth was shown to be essential for increasing daptomycin yield and productivity [6]. However, DA is highly toxic to *S. rosoesporus* and its feeding rate must be kept under strict control in large-scale industrial production [6]. As metabolic engineering efforts continue to increase daptomycin production titers, concomitant with addition of more DA during fermentation, it will be crucial to develop strategies for increasing DA tolerance.

The mechanism of toxicity of free fatty acids (FFA) varies with the length, branching and saturation status of the carbon backbone [7]. The degree of toxicity of a fatty acid also varies across bacteria, with some bacteria being more affected by the length of the carbon backbone while others are more affected by saturation. Their antibacterial mode of action is poorly understood, but most toxicity studies have proposed the cell membrane as the most affected target of fatty acids. In yeast, it has been proposed that DA inserts itself into the lipid bilayer of membrane and physically disturbs the membrane, resulting in increased fluidity of the membrane, leading to conformational changes in membrane proteins, the release of intracellular components [8]. It has been observed that increase of membrane fluidity induced by free fatty acid is accompanied by an increase of ROS production [9]. It can also be hypothesized that the same mechanism may be true for DA.

To elucidate the cytotoxicity mechanism of DA, we combine phospholipid fatty acid analysis, ROS measurement and RNA sequencing technologies to characterize the physiological response to DA and found that resistance to DA likely involves a functional shift of cell membrane composition, increase the gene expression involved in oxidative stress response and oxidative phospholytion. Our findings represent an important advance to understand the mechanism of DA and also provide a list of potential gene targets for further engineering DA tolerance in *S. roseosporus*.

## Results and discussion

### The effect of decanoic acid on the growth of *S. roseosporus*

**Figure 2:**
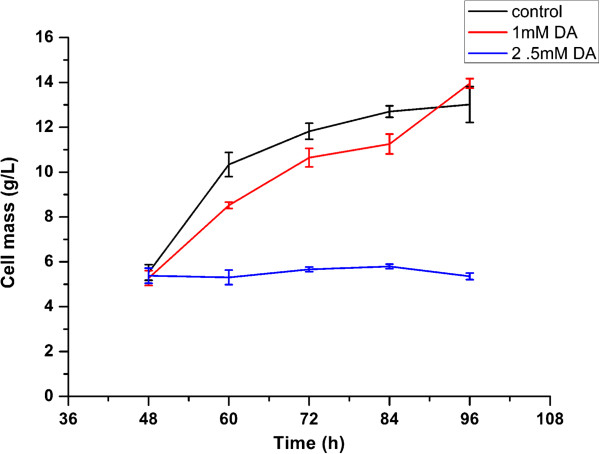
**Cell growth profiles with the addition of 1 mM of decanoic acid at 48 h.** Error bars represent the standard deviation of three biological replicates.

DA was routinely added into the cell culture during the late exponential stage to direct the biosynthesis of daptomycin. In this study, cells were subsequently grown in the presence of a wide range of DA with the FFA added at the late exponential growth phase. Growth of SR was not influenced by 0.5 mM DA. However there was a sharp boundary between sub-inhibitory and growth inhibitory concentrations of DA. A concentration of 1 M caused an approximate 48-52 h lag, followed by normal exponential growth, but a concentration of 2.5 mM halted the growth (Figure 2). The concentration of DA found to be inhibitory to *S. roseosporus* in this study is consistent with that to *S. coelicolor* (2.5 mM).

The concentration of DA that caused stress but not significant cell death was found to be 1 mM and was used in the other growth assay and gene expression analysis.

### Effect of decanoic acid on *S. roseosporus* phospholipid fatty acid composition

**Figure 3:**
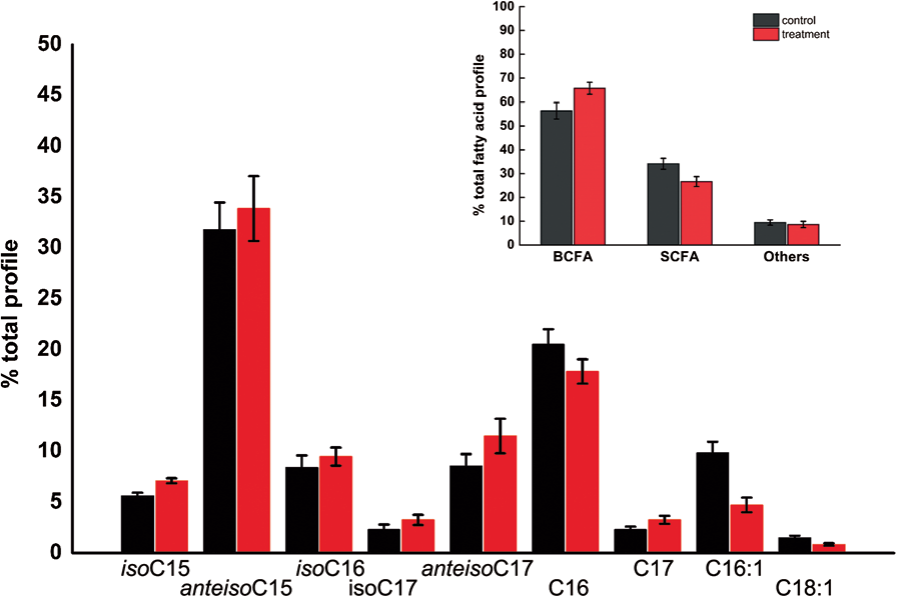
**Phospho-lipid fatty acid profiles of *S. roseosporus *after two hours of decanoic acid-stress.** Control and stressed cell cultures were grown in triplicate. Stressed samples were exposed to1 mM decanoic acid for two hours before being harvested. Cell cultures (50 ml each) were harvested, centrifuged, washed in PBS.

Change in fatty acid profile is associated with FFA adaptation in bacteria [10]. To investigate whether cell membrane reorganization is involved in the DA tolerance, we compare the phospholipid fatty acid profile of *S. roseosporus* cells during late exponential phase growth in either TSB (control), or 1 mM DA. The anteisopentadecanoic (*anteiso*C15), isopalmitic (*iso*C16), palmitic (C16), and pentedecanoic (C16:1) fatty acids made up the majority of total phospholipid composition (Figure 3). During DA stress, the branched-chain Fatty acids (BCFA) dramatically increased, with a corresponding decrease in straight-chain fatty acids (SCFA). Especially, a significant decrease in palmitoleic acid (C16:1) content from approximately 10% to 5% was detectable, implying diminished membrane fluidity. Studies in *Listeria monocytogenes* have demonstrated that the increase of the ratio of BCFA/SCFA plays a significant role in tolerance to acid, temperature, and other stresses by reducing membrane fluidity and decreasing permeability [11]. Similarly, the switch to a fatty acid profile dominated by BCFA suggested a response mechanism leading to a more rigid membrane to mitigate the toxicity of DA.

### Effect of decanoic on the generation of ROS in *S. roseosporus*

**Figure 4:**
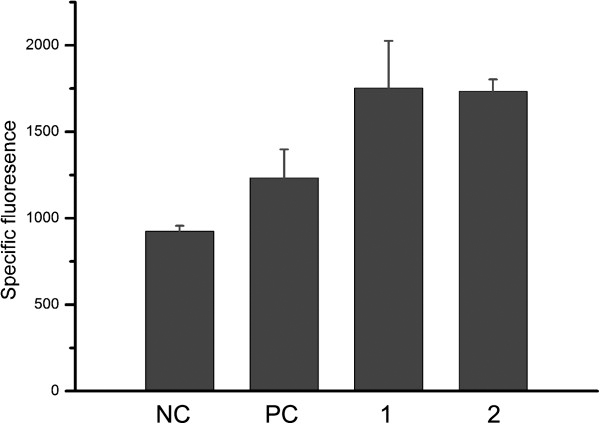
**Measurement of intracellular reactive oxygen species using carboxy-H**_**2**_**DCFDA.** Stressed samples(1, 2) were exposed to 1 mM decanoic acid for 30 min. control cells treated with tertbutyl hydroperoxide (TBHP), known to produce intracellular H_2_O_2_ and serve as a positive control (PC); Cells without treatment serve as a negative control (NC). Measurements were carried out in triplicate.

It has been observed that increase of membrane fluidity induced by FFA is accompanied by an increase of ROS production. To investigate whether ROS involved in the toxicity of DA, we compared the intracellular ROS levels of control and DA-exposed cultures. Cells were labeled with 5(and 6)-carboxy-2’,7’-dichlorodihydro-fluorescein diacetate (carboxy-H_2_DCFDA), a known fluorogenic marker for ROS *in vivo*. Cells exposed to DA showed dramatically high level of fluorescence (Figure 4). Interestingly, cells exposed to different level of DA showed almost the same level of fluorescence, implying that 1 mM DA is enough to induce the ROS generation. These results suggested that exposure to DA may cause massive oxidative stress to *S. roseosporus*.

### Effect of decanoic acid on *S. roseosporus* transcriptome

To elucidate molecular mechanisms underlying tolerance, global gene expression changes during SR growth with DA were analyzed using Illumina RNA deep sequencing (RNA-seq) technology. Tanscriptome libraries were constructed using SR cells grown in the absence (control) or the presence of DA (1 mM).

RNA-seq data revealed a small subset of genes with differential transcription; 134 genes were up-regulated and 12 genes were down-regulated. The presence of DA at 1 mM resulted in transcriptional reprogramming of genes in three major discernible categories, including: energy production and conversion, posttranslational modification and protein turnover, and carbohydrate metabolism (Additional file 1).

### Changes in energy metabolism

Exposure to DA resulted in the up-regulation of genes encoding enzymes proteins (enzymes) involved in energy production and conversion (Table 1). *nuo* operon and *cyd* operon were among the most significantly increased transcripts during DA stress. The *nuo* operon encodes the NADH ubiquinone oxidoreductase (complex I), which form core components of the electron transport chain [12]. The increase in the transcript levels of 12 member of *nuo* operon in response to DA stress suggested that an increased requirement for energy or an impairment in the respiratory efficiency. Increases in the transcripts in the *cyd* operon, which encodes a terminal oxidase and gene (SSGG_06557) encoded the succinate dehydrogenase, both involved in oxidative phosphorylation, also suggested a perturbation in respiratory balance. The up-regulated expression of *nuo* and *cyo* is consistent with the recent discovery that *nuo* and *cyo* played major roles in combating against oxidative stress in *E. coli* exposure to exogenous n-butanol and endogenous fatty acid production [10,13].

**Table 1:** Selected genes involved in energy production and conversion with significant change in expression in DA-stress relative to control

**Locus**	**Gene description**	**Log**_**2 **_**stress/control**
*SSGG_04165*	NuoA	2.55
*SSGG_04166*	NuoB	2.91
*SSGG_04167*	NuoC	3.36
*SSGG_04168*	NuoD	4.09
*SSGG_04169*	NuoE	5.37
*SSGG_04170*	NuoF	3.37
*SSGG_04171*	NuoG	3.07
*SSGG_04172*	NuoH	4.33
*SSGG_04173*	NuoI	2.60
*SSGG_04174*	NuoJ	3.20
*SSGG_04176*	NuoL	2.61
*SSGG_04178*	NuoN	2.91
*SSGG_03413*	CydA	1.20
*SSGG_03414*	CydB	1.19
*SSGG_04984*	F0F1 ATP synthase subunit alpha	1.168
*SSGG_04986*	H(+)-transporting ATP synthase	1.09
*SSGG_06555*	cytochrome b subunit	1.67
*SSGG_06557*	succinate dehydrogenase iron-sulfur subunit	1.37

In addition, the increase in the transcripts in genes encoding the member of the ATP synthase complex (SSGG_04986 and SSGG_04898) was observed. ATP synthase is responsible for generation of ATP through oxidative phosphorylation . It uses energy stored in the pH and potential gradients, created by pumping of protons across the membrance by enzymes of the respiratory chian, to synthesize ATP. Similar results were observed in yeast, where exposure of cells to octanoic acid (C8) led to the activation of plasma membrane H + −ATPase [14]. These results suggested that an increased requirement for energy was required to deal with the DA stress.

### Induction of oxidative stress response

Consistent with results of ROS analysis, the transcriptomic response of *S. roseosporus* to DA showed a massive induction of genes with a role in adaptation to oxidative stress. This response included activation of *clpB* (proteases), *sodF* and several heat shock proteins (Table 2).

**Table 2:** Selected genes involved in oxidative stress response with significant change in expression in DA-stress relative to control

**Locus**	**Gene description**	**Log**_**2 **_**stress/control**
*SSGG_00741*	PafA	1.06
*SSGG_00747*	Pup	1.68
*SSGG_00748*	PafA2	1.52
*SSGG_00750*	Mpa	1.67
*SSGG_01786*	ClpB	1.59
*SSGG_01803*	SodF	1.18
*SSGG_00191*	CepB	2.58
*SSGG_01307*	phage shock protein A	1.45
*SSGG_04333*	Hsp 18	1.65
*SSGG_04334*	hsp60-like protein	1.81
*SSGG_03360*	bifunctional thioredoxin reductase/thioredoxin	1.59
*SSGG_03361*	thioredoxin reductase	2.02
*SSGG_03856*	hsp60-like protein	1.25

In addition, expression levels of genes coding for components of proteasome (*pafA*, *pup*, *pfafA2*, *mpa*) were enhanced. Bacterial proteasomes could only be found in actinomycetes [15]. Mpa assembles into a hexameric ATPase and Pup, a prokaryotic ubiquitin-like protein, is ligated by PafA to substrate proteins. Subsequently, proteins tagged with Pup were targeted for degradation by the proteasome [16]. Recently, it was shown that proteasome is important for defense against reactive nitrogen intermediates (RNI) in *Mycobacterium tuberculosis*[17]. Increased expression of proteasome and clpB may degrade the misfolded proteins impaired by ROS.

In *streptomyces*, the expression of genes coding for proteins involved in antioxidative defense systems was under the control of several key regulators, such as OxyR [18]. However, the homologue of OxyR, master regulator of oxidative response, was absent in *S. roseosporus*. FurS (SSGG_00190) is a zinc-containing redox regulator of *S. reticuli* which binds to an operator upstream of the *furS-cepB*[19]. Under oxidative stress conditions, an internal S-S bridge formed FurS abrogated its capability to block the transcription of *furS-cpeB *[20]. SSGG_00191 shared 80% amino acid identity to *cpeB* of *S. reticuli* and was induced during DA stress. Further studies are required to determine the roles of FurS in oxidative response.

### Changes in carbohydrate transport and metabolism

Transcriptomic analysis suggested a metabolic adaptation dedicated to increase the production of trehalose (Table 3). Expression of genes coding for the putative maltose ABC transporter (SSGG_01377), and Tres (SSGG_05057) which catalyzes the conversion of maltose into Trehalose was elevated. In addition, gene expression of alpha-amylase (SSGG_05058) was induced, which degrade starch to provide the maltose for synthesis of trehalose. The accumulation of this nonreducing sugar has been previously viewed as an important osmoprotectant and stress protectant [21]. It was recently reported that trehalose played an important protective role in the cellular response to oxidative stress caused by hydrogen peroxide in Candida *albicans*[22]. Similarly, *S. roseospous* could counteract the DA stress by increasing trehalose production.

**Table 3:** Selected genes related to carbohydrate metabolism and transport with significant change in expression in DA-stress relative to control

**Locus**	**Gene description**	**Log**_**2 **_**stress/control**
*SSGG_01377*	putative maltose ABC transporter permease	2.46
*SSGG_05057*	trehalose synthase	3.09
*SSGG_05058*	alpha-amylase	2.12
*SSGG_03685*	fructose-bisphosphate aldolase	1.37
*SSGG_06343*	phosphopyruvate hydratase	1.63
*SSGG_01114*	pyruvate kinase	1.30
*SSGG_02477*	phosphopyruvate hydratase	1.53

Genes encoding proteins involved in the TCA cycle were nearly unchanged. Interestingly, expression of genes involved in pyruvate production was up-regulated. Genes coding for fructose-bisphosphate aldolase (SSGG_03685), glyceraldehyde-3-phosphate dehydrogenase (SSGG_06343), phosphopyruvate hydratase (SSGG_02477) and pyruvate kinase (SSGG_01114) were upregulated after exposure to DA stress. Pyruvate is a key intermediate involved in a number of metabolic pathways. It was recently reported that pyruvate is involved into octanoic acid (C8) stress of *E. coli*[23]*.* Addition of pyruvate into media helps the cell partially recover from stress, but the exact mechanism was unclear. Similarly, enhanced production of pyruvate may help *S. roseosporus* to recover from DA stress.

### Preliminary model for the mechanism of DA toxicity and cell response

**Figure 5:**
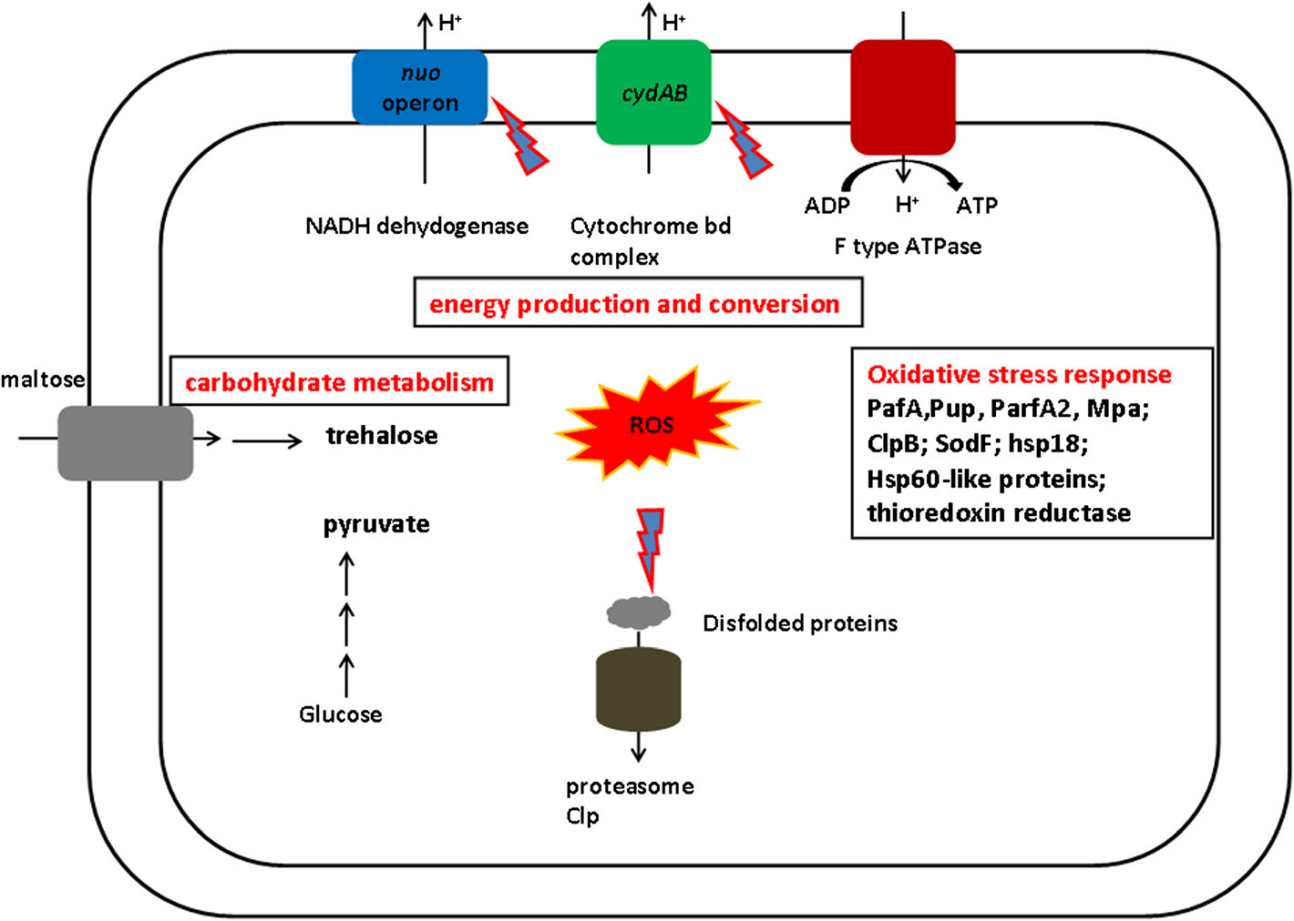
**Illustration about hypothesis about DA toxicity mechanism and the dynamic response of *S. roseosporus*.** DA inserted into the cell membrane disrupted the membrane integrity and induced the generation of ROS by unknown mechanisms. Cells activated expression of a number of genes coding for antioxidative systems to degrade ROS (Superoxide dismutase and catalase), to repair or degrade the disfolded proteins (heat shock proteins, Clp and proteasome). In addition, the yield of stress protectants, such as trehalose may increase to help the cell to mitigate the toxicity of DA.

Based on several lines of evidence, we propose a preliminary model for DA toxicity and cell’s response (Figure 5). DA incorporation into the cell membrane causes the disruption of membrane fluidity. The membrane disruption results in destabililization of membrane-bound proteins, such as NADH dehydrogenase, H-ATPase. Furthermore, DA may directly interact with and partially inhibit of components of electron transport chain, resulting in facilitating electron leakage and generation of ROS. The malfunction of those enzymes will cause inhibition of electron transport chain and lead to less ATP production. To counteract the impaired effect, *S. roseosporus* utilizes multiple ways to protect the cells. Cells increase the ratio of BCFA/SCFA to reduce membrane fluidity and decrease permeability; to protect against the damage caused by oxidative stress, cells activate a number of antioxidant enzymes and repair activities, such as Superoxide dismutase, catalase, and heat shock proteins; furthermore, cells produce high level of trehalose and pyruvate to alleviate the toxicity of ROS. In addition, cells increase the expression of genes encoding proteins involved in oxidative phosphoralation to supply proteins to replace the impaired proteins.

## Conclusions

Multiple mechanisms are involved in the mitigation of the toxicity of DA. The relative contribution of particular mechanism to the toxicity of fatty acid remains elusive. Taken together, our study provided insights into the toxicity or tolerance mechanism underlying DA exposure and several candidates that may be targeted for further engineering to mitigate the toxicity of DA.

## Methods

### Culture conditions

For toxicity assays, *Streptomyces roseosporus* (NRRL11579) was grown in TSB medium. To determine the effect of DA on *S. roseosporus* growth, wide range of concentrations was tested first (data not shown) and then narrowed to a range that caused stress but not significant cell death. Growth assays to test the effect of different concentrations of DA on *S. roseosporus* were performed in 250 ml shake flasks with 25 ml of TSB medium with a 2% inoculation culturing at 200 rpm at 28°C. Unless specified otherwise, all subsequent DA assays were conducted at 1 mM DA.

### Phospholipid fatty acid analysis

Cells were harvested in control culture and DA-stressed *S. rosoeporus* cultures (2 h after exposure to 1 mM DA) of growth by centrifugation at 3000 × g and 4 for 15 min, and the pellet was washed three times with distilled water. The fatty acids in the cells (40–50 mg in wet weight) were saponified and methylated. The methyl ester mixtures were separated using an Agilent 5890 dual tower gas chromatograph. Fatty acids were identified by the MIDI microbial identification system (Sherlock 4.5 microbial identification system) [24]. Minor fatty acids (<0.6% of the total) are not reported.

### Reactive oxygen species assay

Control and DA-stressed *S. rosoeporus* cultures were grown in TSB medium as described with various concentrations of DA. Positive controls for oxidative stressed cells were prepared by adding 10 ul of 7.78 M tert-butyl hydroperoxide (TBHP) (Invitrogen, USA) to one set of control cells before incubation. Ten microliters of 25 mM carboxy-H_2_DCFDA was added to all cells. Florescence at 535 nm was measured after 30 min.

### RNA extraction

*S. roseosprous* was cultured in TSB to exponential phase (48 h). DA was added to a final concentration of 1 mM, and biomass was collected after treatment of 30 min. the cultures were centrifuged at 3000 × g and 4 for 15 min, and cell pellets were immediately frozen in liquid nitrogen and stored at −80 for subsequent RNA isolation. Total RNA was extracted using Trizol (Invitrogen), following manufacturer’s protocols. RNA preparations were treated with RNase-free DNase (Promega) and the integrity of the RNA was determined using Bioanalyzer 2100 (Agilent Technologies). mRNA was enriched by removing the rRNAs using MICROBExpress kit. The mRNA remaining in the supernatant was recovered by ethanol precipitation and quantified by Bioanalyzer 2100. A cDNA library was constructed and sequenced by Illumina Hiseq™ 2000 [25].

### Data processing and analysis

Raw sequencing reads were mapped against the *S. roseosprous* genome. Reads that mapped to more than one region of the genome (5 to 8% of the total) failed to be unambiguously mapped were excluded for subsequent analyses. Analyses of differential expression including FDR calculations were performed using DESeq [26,27]. Only P values of <0.01, FDR ≤ 0.01 were considered to be significant.

## Competing interests

The authors declare that they have no competing interests.

## Authors’ contributions

GL and QL carried out the experiments and analyzed the primary data. GL wrote the draft manuscript. JX supervised the whole work and revised the manuscript. All authors read and approved the final manuscript.

## Supplementary Material

Additional file 1: Table S1 Statistically significant log2 gene expression values for S. roseosporus cultures responding to decanoic acid stress. Click here for file
